# Progress in Skin Diseases

**Published:** 1903-03-28

**Authors:** 


(*To be continued.*)

### Progress in Skin Diseases.

**Dermatitis Herpetiformis.**—R. Park[1] reports a remarkable case which was cured by daily injections of adrenalin. The patient suffered also from pyonephrosis and pyosalpinx for which she underwent operation. The herpetiform eruption covered a large part of the trunk and limbs, and the general condition was serious with a rapid, weak pulse. One minim of Parke Davis and Company's solution of adrenalin was injected hypodermically and this was gradually increased to 20 minims daily with remarkable success. The skin affection entirely disappeared and the pulse became normal. Whenever the injections were omitted the eruption returned, and as the full dose caused faintness, after a time it was gradually reduced to 5 minims daily, which she continued to take up to the time of writing, when the skin appeared to be perfectly healthy.

**Von Recklinghausen's Disease.**—Nuttall and Billington[2] report a case of general neurofibromatosis with a necropsy. There was a history of a wound on the right cheek at three years of age. Two years later a lump appeared which was removed six years after, but the swelling continued growing. At 23 years there was a freely movable tumour in the same cheek with a string of small ones running back to the angle of the jaw, and numerous others on the trunk and some on the forehead and right arm. The large tumour was removed and the patient remained well for 14 years, when it again began to grow and the whole body was found to be covered with small nodules for the most part painless and without interference with the functions of the nerves on which they were placed. The skin was dry, coarse, and of a bronzed appearance. The patient died after an operation for the removal of a tumour from the mouth which interfered with swallowing. Each of the tumours was connected with a nerve which ran outside it in one or more bands and could be easily separated from it. The nerves showed fusiform swellings at intervals. Besides the peripheral nerves the phrenic, vagi, and splanchnic were similarly nodulated. To every tumour not actually in the skin a nerve could be traced. The disease has been looked upon as a vice of development, but in this and other cases there is a clear history of an injury.

Seborrhœa.—Norman Walker[3] points out that the name has been generally applied to the scaly form, and that most writers look on the scales as the excretion of the sebaceous glands. However it is probable that much of this greasy matter comes from the sweat glands. If seborrhœa exists only in the scaly form baldness does not occur, but there is often premature greyness. The connection between seborrhœa of the scalp and that of the body is a difficult one, and the relations between seborrhœa and psoriasis even more hard to make out. As to treatment, sulphur which is useful in the moist forms of the disease has little effect in the drier ones. Leslie Roberts remarks that the fat excreted by the sweat glands is a fluid oil, that of the sebaceous glands is an exfoliation. He would confine the term seborrhœa to the affection of the latter type. Sabouraud's bacillus has failed to produce the disease when inoculated and no one has proved that it produces any toxin. Where the sebaceous glands are active the skin is fertile in bacteria, but their importance is an uncertain matter. The excess of the secretion itself leads, however, to various forms of disease, such as acne, and some forms of pityriasis, eczema, and baldness. J. A. Ormerod[4] gives a clear account of the opposing theories of the disease. Sabouraud, he says, recognises only the oily form as seborrhœa. This is caused by his special bacillus which produces a fatty plug in the sebaceous glands, and may lead to other complications such as pustules or baldness. The dry form is really a pityriasis, and due to other causes. Unna, on the other hand, thinks both forms are due to the oily state of the skin, and that both the sebaceous and the sweat glands supply this material. It is said, too, that the scaly form is due to the breaking up of the cells of the sebaceous glands. If an eczema occurs in an oily skin of this type, Unna thinks that the ordinary intestical œdema between the cells leading to the formation of vesicles, is prevented, and this brings about a dry scaly papular eruption which is known as seborrheic eczema. Schamberg[5] found the bacillus of seborrhea in the nasal follicles of 45 out of 50 persons. In 16 of the 45 the skin was dry, and in 27 it was oily. He thinks if this organism is so extremely common in healthy individuals there can be little reason for regarding it as the special cause of the disease. Radcliffe Crocker[6] draws attention to the forms of dermatitis which occur on a seborrhœic skin. His first group includes (*a*) An eczematous form, dry, scaly, attacking the neck, chest, back, axillæ, and groin, in rounded patches; (*b*) a papular type forming circles of close-set papules on the centre and upper part of the chest and back; (*c*) a psoriasiform type attacking chiefly the trunk instead of the limbs, and showing patches less definitely scaly and circular than those of true psoriasis. When this form attacks the scalp there is always much falling out of the hair, which does not occur in true psoriasis. His second group includes pustular eruptions, such as chronic furunculosis, sycosis, acne keloid, etc., and here the pus organisms must be got rid of as well as the seborrhœa. This, however, is often difficult, for the pus cocci in these cases seem to go very deeply, and their destruction requires constant and persevering treatment.

1 Scottish Med. and Surg. Jour., Nov. 2 Lancet, Dec. 27. 3 Brit. Med. Jour., Oct. 25. 4 Clin. Jour., Vol. XX., No. 18. 5 Jour. Cut. and Gen.-U. Dis., 1902. 6 Lancet, Feb. 21.

